# A systematic review of the methodology for examining the relationship between obstructive sleep apnea and type two diabetes mellitus

**DOI:** 10.3389/fendo.2024.1373919

**Published:** 2024-09-04

**Authors:** Manal Taimah, Nirmin F. Juber, Paula Holland, Heather Brown

**Affiliations:** ^1^ Division of Health Research, Lancaster University, Lancaster, United Kingdom; ^2^ Public Health Research Center, New York University Abu Dhabi, Abu Dhabi, United Arab Emirates

**Keywords:** methodology, obstructive sleep apnea, diabetes, disparities, adults, systematic review

## Abstract

**Background:**

The association between obstructive sleep apnea (OSA) and type 2 diabetes mellitus (T2DM) has been explored in various studies, revealing inconsistent correlations that impact therapeutic effectiveness. This heterogeneity in findings requires further exploration to understand what may be driving this. Therefore, this study focuses on systematically reviewing the data, classification of variables, and analytical approach to understand if and how this may be contributing to the mixed findings. This review aims to provide insights that can enhance the generalisability of future research findings.

**Methods:**

A comprehensive electronic search was conducted, including EMBASE, MEDLINE, PsycINFO, CINAHL, Web of Science Core Collection, Scopus and specialised sleep journals. The included studies were observational studies published in English from 2011 onwards, involving adults above 18 years with OSA and T2DM or prediabetes, and included a control group. Exclusions were pregnant women, interventional studies, randomised trials, systematic reviews, conference abstracts, case studies and studies without a control group or only with descriptive analysis.

**Results:**

We reviewed 23 studies that met the inclusion criteria. Among cohort studies, 54% did not report attrition rates, and 52% did not detail methods for handling missing data in all studies. Nine studies (39%) predominantly included male participants. Objective measures were prevalent in assessing OSA, with 11 using home portable sleep monitors and four employing clinic polysomnography, though only three validated home sleep monitors. The apnea-hypopnea index was commonly used to define OSA severity, with six studies adapting the American Academy of Sleep Medicine criteria. Two studies utilised validated self-report questionnaires for OSA symptoms. T2DM diagnosis methods varied, with 17 studies using blood samples, two relying only on self-reporting, and four confirmed diagnosis via medical records.

**Conclusions:**

The variability in sample characteristics, data quality, and variable coding may contribute to the mixed finding. This review identifies gaps in using the standardised measures, reporting attrition rates, handling missing data, and including both sexes. Addressing these issues is crucial to enhancing future research generalisability. Standardising diagnostic criteria, considering clinical and sociodemographic factors, and ensuring inclusivity in study populations are essential for advancing understanding and treatment strategies for OSA and T2DM.

**Protocol registration:**

https://www.crd.york.ac.uk/prospero, identifier CRD42023397547.

## Introduction

Obstructive sleep apnea (OSA) is a commonly underdiagnosed sleep disorder characterised by a recurrent episode of partial or complete upper-airway collapse causing cessation in ventilation, sleep apnea, hypoxia, and fragmented sleep (1, 2). OSA affects almost one billion adults globally, with one in four men and one in nine women being affected (3). The severity and symptoms of OSA can vary significantly between different ages and sexes (4, 5). Individuals with OSA may have an increased risk of having chronic diseases, such as type two diabetes mellitus (T2DM), and may experience poor health outcomes and disease prognosis (2, 6, 7).

There have been several cohort studies conducted to investigate the link between OSA and T2DM (8–14). However, the results of these studies have been inconclusive and have provided mixed findings. Out of the studies conducted, some cohort studies have found that only moderate to severe OSA increases the risk of developing T2DM (9, 10, 13, 14). A cohort study showed that mild OSA is also directly associated with the incidence of T2DM (8). However, other cohort studies revealed no significant association between OSA and T2DM (11, 12, 15).

Although several cohort studies have evaluated the correlation between OSA and T2DM, their methodology has significant differences (11, 16–18). For instance, the age range of participants varied across studies, with some studies including individuals between 43 and 61 years old and others only including those above 70 years old (11, 13). Additionally, the methods used to diagnose OSA also varied between studies. Some cohort studies used self-reported symptoms, while others used clinic polysomnography (PSG) assessment for OSA diagnosis (15, 16, 19–21). These differences in methodological approach and population characteristics may account for the inconsistencies in the reported association between OSA and T2DM.

This systematic review aims to explore the approaches, data collection methods, variable classification and statistical techniques used in studies of OSA and T2DM to understand how these factors may contribute to the mixed findings. By providing insights into these methodologies, this review seeks to enhance the generalisability of future research findings.

Previous systematic reviews assessing the OSA and T2DM association have often focused on pooled risk assessments without thoroughly examining the methodological approaches, including sample characteristics, variable coding, and analytical approach among primary studies (7, 22). Therefore, this review focuses on the methods of primary studies to understand what drives the mixed findings observed in epidemiological studies regarding the OSA and T2DM correlation. Understanding differences in study design, participant characteristics, and assessment tools will provide insights into how these factors influence the reported associations between OSA and T2DM. This understanding is crucial for improving the interpretation and generalisability of research findings, guiding future studies to adopt more standardised approaches to what information is reported, and ultimately enhancing the evidence base for clinical decision-making and policy development related to OSA and T2DM (4, 23).

### Review question

What are the different methodologies used to investigate the association between OSA and T2DM in adults? How do these methodologies impact the strength and nature of the association?

#### Objective

The main objectives of this review are to:

1. Understand the methods, procedures, and statistical tests used to evaluate the association between OSA and T2DM.
2. Understand the strengths and weaknesses in the methods used and how this may affect understanding the association between OSA and T2DM results.
3. Identify gaps in knowledge and suggest possible areas of development and research.

## Methods

The protocol of this systematic review was registered and published with PROSPERO CRD42023397547 and conducted according to the Preferred Reporting Items for Systematic Review and Meta-Analyses (PRISMA) statement (24).

### Search strategy

One researcher (M.T.) with a librarian specialist completed a systematic literature search to identify all original research articles published in English and report the association between OSA and T2DM in the adult population between January 1, 2011, and March 30, 2023. The electronic search was conducted in six databases, including EMBASE, MEDLINE, PsycINFO, and CINAHL, through the EBSCO database, Web of Science Core Collection, and Scopus. The specialised journals in sleep medicine and diabetes, including Sleep Journal, Journal of Clinical Sleep Medicine, and Journal of Sleep Research, were also searched manually to identify possible publications that could be used in the review. Further, the bibliography list of the included articles was also checked for additional references to avoid missing eligible articles that can be included in this review.

The search terms were located and extracted from multiple sources, including abstract keywords, research papers and previous systematic reviews on the area of OSA and T2DM. The terms were listed in a table and checked on the selected databases. The key search terms were Sleep Apnea, Obstructive, Sleep Apnea Syndromes, Diabetes Mellitus Type 2, and Epidemiologic Research Design. The detailed search strategy, including the list of search terms and the specific queries for each database search, is provided in the **Supplementary File 1: Appendix A**.

Each search term was entered into the Mesh search database for the MEDLINE database, and the corresponding Mesh term was checked. The selected Mesh term was also expanded, and the related Entry terms were checked to verify an alternative form of the entered search term that could be used interchangeably in the literature. The identified Mesh terms were added to the search query. Some Mesh terms were not expanded if the subheading terms were unrelated to the research question. The search query included a mix of mesh and free text terms to maintain a comprehensive search strategy for each concept. The APA PsycINFO database was searched using the APA Thesaurus of Psychological Index Terms. The heading terms were expanded to include subheading terms related to the concepts. Other free text terms were included in the search query to extract all the related articles. For the CINAHL database, the CINAHL Subject Headings were searched first to find the key searching terms, and the other text terms were added to locate all related papers.

The equivalent search process was used for Scopus and Web of Science Core Collection databases by inserting the keyword in the advanced search page and combining each concept results to find the papers related to the research question. EMBASE database was searched via the Ovid database. The Map Term to Subject Heading option was selected to map the key terms to the subject heading in the database to find all the alternative concepts that can be used to search for the papers. The Ovid thesauruses explode option was selected after checking the sub terms. Free text terms were also combined with the database thesauruses to retrieve all the related research papers.

### Eligibility criteria

The eligibility of all located studies was assessed using a PECO framework (Population, exposure, comparison and outcome), as shown in **Table 1** (25). The inclusion criteria encompassed adult individuals aged 18 years and over from diverse racial and ethnic backgrounds who have OSA and T2DM or prediabetes. The review focused on original observational studies published in English between January 1, 2011, and March 30, 2023 prioritising the most recent publications when multiple studies utilised the same dataset and had similar objectives. Prioritising the most recent publications ensures the use of updated methods, refined data analysis in response to peer feedback, and relevance of the results to current practices. Eligible studies were required to include a control group free of either condition (T2DM or OSA).

**Table 1:** The PECO framework for the Eligibility criteria.

PECO framework	Description of the inclusion criteria	Exclusion criteria
**Population**	Adult individuals aged 18 years and above of both sexes and different races or ethnicities, using original studies published in the English language between January 1, 2011, and March 30, 2023	Pregnant participants, due to the physiological effect of pregnancy on sleep, participants under 18 years. Studies have not been published in the English language.
**Exposure**	Patients with OSA and T2DM who participated in observational studies. The most recent publication will be included when multiple publications using the same dataset and similar aims are found	Interventional studies since the focus is to understand the methods used to understand the link between OSA and T2DM. Randomised clinical trials, Systematic reviews, conference abstracts, and case reports
**Comparison**	Compare the different methods, assessment procedures, and statistical analysis. Eligible studies must include a comparative group that has no disease (control group free of OSA or T2DM)	Studies that only included descriptive analysis and prevalence were excluded. Studies without a control group
**Outcome**	The methods used to understand the association between OSA and T2DM	This review will not address the prevalence of OSA and T2DM or the strength of their association

OSA, obstructive sleep apnea; T2DM, type 2 diabetes mellitus.

The exclusion criteria for this review were as follows: Studies involving only participants below 18 years old or pregnant women due to the physiological effects of pregnancy on sleep. Studies not published in English were excluded due to limited resources for translation. Interventional studies were not considered, as the focus was on understanding the epidemiological evidence used to investigate the link between OSA and T2DM. Randomised clinical trials were excluded because we are not evaluating interventions. Additionally, systematic reviews, conference abstracts, and case reports were excluded. Studies that included only descriptive analysis and prevalence without a control group were also excluded to ensure the inclusion of research offering robust comparative data essential for understanding associations and causality between OSA and T2DM. Lastly, this review did not address the prevalence of OSA and T2DM or the strength of their association since the focus is to understand the methodological approaches. Two researchers (M.T. and N.J.) screened the title and abstract, and the disagreement was resolved by discussion. Eligible identified studies were moved for full-text review.

This review prioritises accuracy and comparability by focusing on studies published from 2011 onwards, ensuring the inclusion of research employing the most current diagnostic criteria for both OSA and T2DM. The American Academy of Sleep Medicine revised its scoring manual in 2007, refining criteria for respiratory events to improve the consistency and accuracy of OSA assessments (26). Additionally, in 2010, the American Diabetes Association incorporated the haemoglobin A1C test (HbA1c ≥ 6.5%) as a standard diagnostic tool for T2DM, alongside existing measures such as fasting blood glucose and oral glucose tolerance tests (27). With the widespread adoption of these updated guidelines, studies published from 2012 onwards are more likely to reflect these practices, enhancing the comparability and reliability of the findings. By focusing on post-2010 studies, this review ensures adherence to the latest diagnostic standards, thereby strengthening its findings on the association between OSA and T2DM (26–29).

### Full-text review and data extraction

The full-text review of all the included studies was independently completed primarily by M.T and partially by N.J. In order to address the review question with precision, it is essential to gather all the necessary information from the included studies using a well-developed extraction tool that aids in the collection of important data (30, 31). An extraction template was created by M.T and reviewed by H.B and P.H, using extraction tools employed in previous systematic reviews to complete the extraction step (22, 32). The extraction template was integrated into the Covidence software to ensure a seamless and effective extraction process (33). Two researchers (M.T. and N.J.) tested the applicability of the extraction template and reviewed the data extraction agreement using five studies before starting the actual data extraction process (data were excluded and re-performed). Eligible studies were assessed on information including study title and author details; study population and setting (e.g., demographic characteristic and study country and completion rate); comparator variables; details on study methodology (e.g., study design, sampling method, time, statistical method, analysis framework); study outcomes, information of the risk of bias assessment and missing data treatment.

In this review, one eligibility criterion is to have a control group of individuals without the condition of interest, namely OSA or T2DM (34). Two distinct comparator groups were included: The control and matched control groups (34). The control group was not specifically matched and was selected based on assessment results (34). For instance, individuals who did not exhibit symptoms of OSA were chosen as the comparator group, or those who did not have T2DM at the onset of the study were compared to those who did to gauge the time differential in developing OSA. On the other hand, a matched control group involves selecting individuals who match the disease group with respect to specific variables (34).

We collected data on the sampling frame and sampling method. When conducting research utilising primary or secondary data, it is imperative to take into account the sampling frame and sampling method. The sampling frame is a comprehensive list of the population of interest to obtain a selected sub-sample (35). For instance, if medical records of specific patients are being used, the list of those medical records would serve as the sampling frame. While sampling methods include the description of sampling techniques used by the researchers to obtain the sample of the participants, these techniques can be probability and nonprobability (35).

This review examines previous studies investigating the relationship between OSA and T2DM, presenting three primary hypotheses. The first hypothesis suggests that OSA may contribute to the development of T2DM, while the second hypothesis proposes that T2DM may increase the risk of developing OSA. The third hypothesis suggests that there may be a bidirectional association between the two conditions. In case the reports of the studies lacked important information, we emailed the authors of the respective studies to request the missing information. The consensus between the two researchers on data extraction was reviewed in seven included articles, and the discrepancies were solved through discussion. All included articles were extracted by M.T and verified by N.J. A third author (H.B.) was consulted if there were unsolved issues.

### Assessment of study quality

The quality of the included studies was assessed using two different forms, the Newcastle-Ottawa Quality Assessment Form for cohort studies and the Joanna Briggs Institute checklist for cross-sectional studies (see **Supplementary File 2: Appendix B**) (19, 36, 37). Before completing the appraisal process, two researchers independently tested the process using five studies (three cohort studies and two cross-sectional studies) to assess the agreement and applicability of the appraisal tools. Data were excluded and re-performed. The quality of all the included articles was appraised primarily by M.T and reviewed by N.J, and the discrepancies were solved through discussion. A third author (H.B.) was consulted if there were unsolved issues.

### Data synthesis

This review utilised the checklist of the Preferred Reporting Items for Systematic Reviews and Meta-Analyses (PRISMA) statements to assess the robustness of the systematic review report (see **Supplementary File 3: Appendix C**). This step involved thoroughly evaluating the review process, ensuring that all necessary components for a systematic review were adequately addressed. Adhering to PRISMA guidelines enhanced the review’s transparency, reproducibility, and comprehensiveness (24). Before starting data synthesis, we explored the heterogeneity within studies. We found extensive heterogeneity (see **Supplementary File 4: Appendix D**). Thus, we employed a narrative approach following the adapted guidelines outlined by Popay and colleagues (38):

For our narrative synthesis, first, we begin by presenting the data in tables, allowing for a clear presentation of the findings. Each study’s key characteristics—such as objectives, study design, sample characteristics, definitions of OSA and diabetes, and methodological approaches, including handling of missing data—are systematically detailed to facilitate comparison and contrast.

Step 2 involves exploring relationships between different study types and their findings, examining how varied study characteristics, contexts, and methodological approaches might influence outcomes. For instance, demographic data from participants were extracted to assess their potential impact on understanding the association between OSA and T2DM and the generalisability of results. This structured approach aided in comprehending current methodologies and their suitability in investigating the relationship between OSA and T2DM.

Step 3 Assessing the Robustness of the Synthesis. The synthesis process involves evaluating the strength of the findings by considering the quality of the included studies, the consistency of findings across studies, and the extent to which findings are backed by evidence. This step was completed by assessing the included studies’ quality using the Newcastle-Ottawa Quality Assessment Form for cohort studies and the Joanna Briggs Institute checklist for cross-sectional studies (36, 37).

Finally, consider the implications of the findings and write the synthesis. This was completed by discussing the practical implications of the synthesised findings for policy, practice, and further research, writing up the narrative synthesis, and presenting the findings in a coherent and structured manner.

## Results

**Figure 1:**
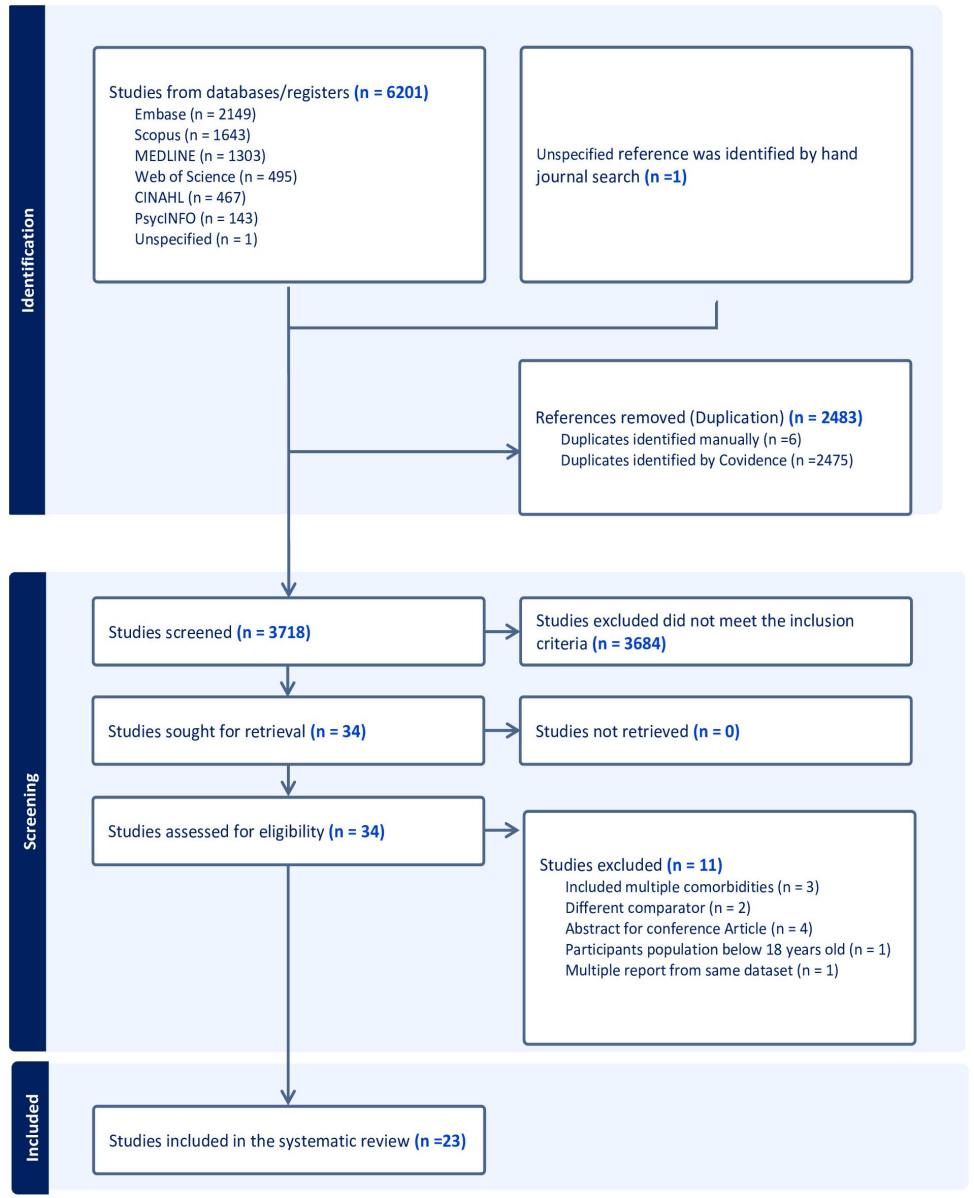
The PRISMA Flow Diagram of the study inclusion process.

A total of 6210 studies were found in the designated databases, and an additional article was discovered through a manual search. After removing duplicate publications found across multiple databases, 3718 distinct articles underwent screening of their title and abstracts. Following this preliminary screening, 3684 articles were deemed irrelevant, and 34 relevant studies were selected for full-text review. During the full-text screening, 11 studies were excluded based on the set inclusion and exclusion criteria, leading to a final selection of 23 articles for review. The PRISMA flow chart (**Figure 1**) presents details on the search and review process (24).

### Study characteristics

**Table 2** shows the characteristics of the included studies. This systematic review included 23 studies published between February 2012 and April 2023. Two main study designs were identified in the current review: cohort studies (56.5%, n=13) (8, 10–14, 18, 39–44) and cross-sectional studies (43.4%, n=10) (5, 17, 45–52). Out of the thirteen cohort studies, six were retrospective, and seven were prospective (see **Table 2**).

**Table 2:** Summary of the characteristics of the review studies.

Author/year	Study design	Setting	Sample information	Sample size	Mean age	Male %	Number of groups	Factors adjustment	Quality assessment
1 KorshÃ¸j 2020 (49)	Cross-sectional study	Denmark	Active recruitment	57	49.3 ± 7.8	100%	Two groups: OSA, No-OSA	Age, level of leisure time and physical activity	Fair
2 Harada 2012 (17)	Cross-sectional study	Japan	Active recruitment	275	44 ± 8	100%	Four groups: T2DM with NFG, IFG with NFG	Age, waist circumference, RDI, average sleep fragmentation, ESS scores and sleep duration	Good
3 Lindberg 2012 (18)	Prospective cohort study	Sweden	Convenient previous study	141	57.5	100%	Two groups: T2DM, No-T2DM	Age, BMI, and hypertension at baseline and BMI and years with CPAP treatment	Good
4 Leong 2014 (50)	Cross-sectional study	United Kingdome	Convenient medical record	283	47.1 ± 12.1	33.2%	Two groups: T2DM, No-T2DM	Age, sex, BMI, ethnicity, and number of DM medications	Fair
5 Vacelet 2021 (12)	Prospective cohort study	France	Convenient medical record	494	66.2 ± 0.9	Mild OSA: 29%, Moderate OSA: 42.1%, Severe OSA: 60%.	Three groups: Mild OSA, Moderate OSA and Severe OSA	Sex, BMI, fat mass, fasting glucose, and triglyceride	Good
6 Appleton 2015 (11)	Prospective cohort study	Australia	Convenient previous study	736	59.7	100%	Three groups: Mild OSA, Moderate OSA and Severe OSA	Age, education, income, total percentage of body fat, smoking, physical activity, weight gain over the follow-up period, and follow-up measures including ESS scores, sleep hours, and shift work	Good
7 Ding 2021 (8)	Retrospective cohort study	United States of America	Convenient previous study	840	57.6 ± 12.4	94.4%	Two groups: T2DM, No-T2DM	Sex, age, race/ethnicity, baseline fasting glucose concentrations, BMI, changes in BMI over time, hypertension, heart failure, myocardial infarction, depression, smoking status, and alcohol use	Good
8 Deol 2018 (46)	Cross-sectional study	United States of America	Convenient previous study	899	55 ± 9	54%	Three groups: NFG, pre-DM, T2DM	Age, sex, site, residency years, income and education, smoking, alcohol use, waist, blood pressure, and BMI	Good
9 Sanchez 2022 (51)	Cross-sectional study	Spain	Convenient previous study	966	Pre-DM, 59; Control, 56	Pre-DM=42.2% Control =51,8%	Two groups: NFG, pre-DM	Age, sex, and BMI	Good
10 Xu 2019 (10)	Retrospective cohort study	China	Convenient medical record	1206	51	69%	Four groups: No-OSA, Mild OSA, Moderate OSA, Severe OSA	Age, sex, BMI, body weight change, waist circumference, cigarette smoking, alcohol use, family history of T2DM, ESS score, prior Comorbidities, physical activity, neck circumference, and total sleep time	Good
11 Ali 2023 (14)	Retrospective cohort study	Korea	Convenient previous study	1216	57.5 ± 5.5	46.9%	Three groups: No-OSA, Mild OSA, Moderate to severe OSA	Age, sex, occupation, income, waist, waist change, exercise, alcohol, smoking, mean arterial pressure, cholesterol, cardiovascular disease history, total sleep time and napping	Good
12 Kim 2013 (48)	Cross-sectional study	Korea	Convenient previous study	1344	40 ± 69	52.5%	2 groups: BMI <25 kg/m2, BMI ≥ 25 kg/m2	Age, sex, alcohol, smoking status, exercise, hypertension or cardiovascular diseases, and medication for dyslipidaemia, BMI, and waist circumference	Good
13 Nagayoshi 2016 (41)	Prospective cohort study	United States of America	Convenient previous study	1453	62.5	46.5%	Three groups: No-OSA, Mild OSA, Moderate to severe OSA	Age, sex, centre, education, income, occupation, marital, smoking, alcohol, physical activity, BMI, and waist circumference	Good
14 Whitaker 2018 (52)	Cross-sectional study	United States of America	Convenient previous study	2049	68.5 ± 9.2	46.5%	Two groups: T2DM, No- T2DM	Centre, age, ethnicity, sex, education, marital status, smoking, alcohol, hypnotic medication, depressive symptoms, BMI, and waist circumference	Good
15 Beate Strand 2015 (39)	Prospective cohort study	United States of America	Convenient medical record	5888	72	Unclear	Two groups: day sleepiness, No day sleepiness	Age, sex, race, waist circumference, site, marital status, education, smoking, alcohol BMI, physical activity, depressive symptoms score, cognitive function, systolic blood pressure, antihypertensive medication, creatinine, albumin, cholesterol, history of myocardial infarction	Fair
16 Sabanayagam 2012 (5)	Cross-sectional study	United States of America	Convenient previous study	6522	no DM 43.7 ± 0.4; DM 55.4 ± 0.6	51.2%	Two groups: T2DM, No-T2DM	Age, sex, race-ethnicity, education, smoking, current alcohol consumption, physical activity, BMI, systolic blood pressure, depression, CRP, cholesterol	Good
17 Kent 2014 (47)	Cross-sectional study	Europe	Convenient previous study	6616	52.1± 12.3	70.7%	Four groups: No-OSA, Mild OSA, Moderate OSA, Severe OSA	site, race, age, sex, BMI, neck circumference, smoking, alcohol, heart and lung comorbidities, medication use (B-blockers, oral corticosteroids, and statins)	Fair
18 D’Aurea 2017 (45)	Cross-sectional study	Brazil	Convenient medical record	7115	43.4 ± 9.6	57.6%	2 groups CRP < 2, CRP ≥ 2 and by HbA1C <5.7% HbA1C ≥5.7%	Age, sex, systolic blood pressure, waist circumference, high-density lipoprotein, cholesterol, and triglycerides	Fair
19 Kendzerska 2014 (13)	Prospective cohort study	Canada	Convenient previous study	8678	48	62%	Two groups: T2DM, No-T2DM	Sex, age, BMI, history of smoking status, prior comorbidities, daytime sleepiness, neck circumference, heart rate in sleep, and total sleep time	Good
20 Strausz 2018 (42)	Prospective cohort study	Finland	Convenient previous study	36963	FINRISK data, 48.01 ± 13.2; Health 2000 data, 53.8 ± 15.7 Botnia data: 58.94 ± 11.5	FINRISK data, 47.6%; Health 2000 data, 44.6%; Botnia data:52.3%	Two groups: OSA, No-OSA	Age, sex, geographical area, BMI and cohort year, high-density lipoprotein and total cholesterol, smoking, family history of heart disease, hypertension	Good
21 Liu 2017 (40)	Retrospective cohort study	Taiwan	Convenient medical record	358967	Unclear	Analysis 1, 64.2%; Analysis 2, 52.5%	Four groups (analysis I): OSA, No-OSA. (analysis II): T2DM, No-T2DM	Age, sex, hypertension, hyperlipidaemia, congestive heart failure, cerebrovascular disease, use of cardiometabolic agents, including B-blockers, calcium channel blockers, angiotensin-converting enzyme inhibitors, angiotensin receptor blockers or lipid-lowering agents	Good
22 Tianyi 2018 (44)	Retrospective cohort study	United States of America	Convenient previous study	146519	The Nurses Health Study, 69.9; The Nurses Health Study II, 51.4; The Health Professionals Follow-up, 68.8	The Nurses Health Study, No males The Nurses Health Study II, NO males; The Health Professionals Follow-up Study, 100% males	Four groups (analysis I): OSA, No-OSA. (analysis II): T2DM, No-T2DM	Age, race, menopausal, family history of diabetes, duration of postmenopausal hormone use, smoking status, alcohol, diet quality, regular physical examination, sleep duration, night shift duration, physical activity, hypertension history, BMI, and waist circumference	Good
23 Subramanian 2019 (43)	Retrospective cohort study	United Kingdome	Convenient medical record	1656739	Exposed group, 64.85 ± 13.28; Unexposed group, 64.56 ± 13.63	Exposed group: 55.5% Control group: 54.2%	Two groups: T2DM, No-T2DM	Age category, sex, BMI category, Townsend deprivation quintile, smoking status, ethnicity, and baseline cardiovascular conditions (heart failure, ischemic heart disease, stroke/transient ischemic attack, atrial fibrillation, and hypertension)	Good

T2DM, type 2 diabetes mellitus; NFG, normal fasting glucose; IFG, impaired fasting glucose; RDI, respiratory disturbance index; ESS, Epworth sleepiness scale; Pre-DM, prediabetes; CPAP, continuous positive airway pressure; CRP, C-reactive protein; HbA1C, Haemoglobin A1C.

**Figure 2:**
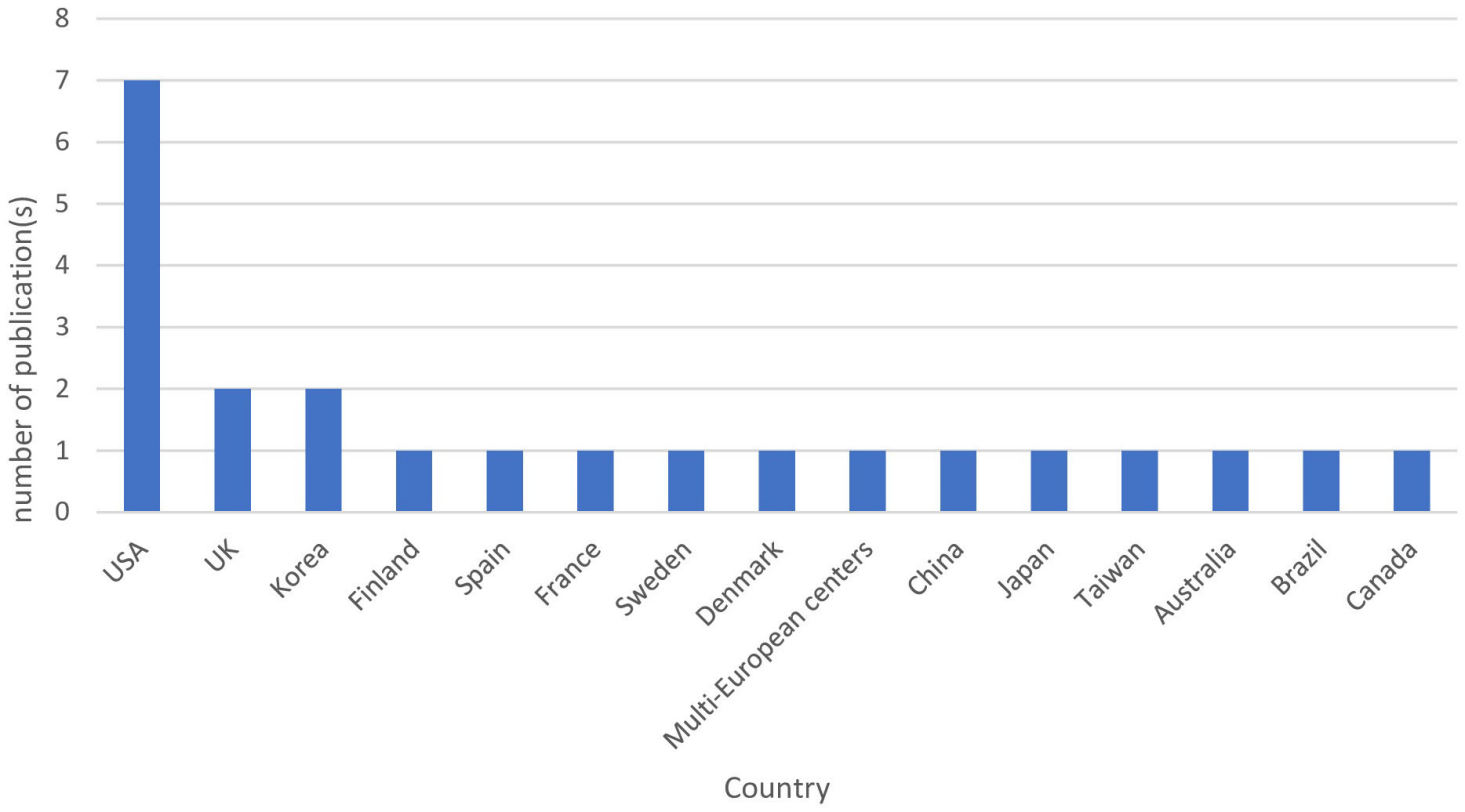
Geographical distribution of the studies included in the review.

**Figure 2** represents the geographical distribution of published studies. The included studies represent 15 different countries, and most of the studies were from European countries (34.8%, n=8 studies), including the United Kingdom, Spain, France, Sweden, Denmark, and Finland (12, 18, 42, 43, 47, 49–51). This was followed by the United States of America (30.4%, n=7 studies) (5, 8, 39, 41, 44, 46, 52). Asian countries contributed by five studies, including China, Japan, Taiwan, and Korea (21.7%) (10, 14, 17, 40, 48). Canada (13), Brazil (45), and Australia (11) each contributed by one study. This review highlights the diverse geographic representation of the included studies, with a notable concentration in English-speaking countries, while regions such as the Middle East and Africa were not prominently represented.The primary aims of the included studies were to investigate the effect of OSA in the incidence of T2DM (39%, n=9), influence of OSA and sleep variables on glucose metabolism and T2DM control (34.8%, n=8), impact of T2DM and OSA on their respective incidences (8.7%, n=2), and the effects of T2DM on the variation in OSA and the risk for OSA (8.7%, n=2). One study focused on the impact of T2DM on the incidence of OSA (4.3%, n=1), while another aimed to determine the prevalence and risk factors of OSA with T2DM (4.3%, n=1).

### Methodology characteristics

**Table 2** shows information about the sampling method and the sampling frame of the included studies. In this review, two cross-sectional studies were conducted using primary data that actively recruited a non-random sample of participants (17, 49). Of the other 21 studies, 14 utilised previously collected data from other studies, and seven relied on administrative data from patient medical records. One cohort study by Liu and colleagues randomly selected a sample of patients from a health insurance database and included them in the analysis (40).

#### Sample characteristics

The current review showed a wide variation in sample size (see **Table 2**). The 23 reviewed articles recruited a total of 1,656,739 participants with a sample size ranging from 57 to 1,656,739 (43, 49). In this review, four studies exclusively enrolled male participants, and five studies used mainly men who accounted for 60% or more of the sample (8, 10, 11, 13, 17, 18, 40, 47, 49). One study by Beate Strand and colleagues did not specify the sex ratio of their participants (39). In the study by Tianyi et al., they included two datasets, each containing data for only one gender: one dataset included 100% men, and the other included 100% women (44). The remaining 12 studies included both sexes with females accounting for 40% or more of the sample and reported the percentage of male and female participants (see **Table 2**) (5, 12, 14, 41–43, 45, 46, 48, 50–52).

Within the included studies, 20 studies did not calculate the required statistically significant sample size, while three studies utilised pre-existing samples based on power calculations to determine the required sample size (5, 8, 13). The inclusion and exclusion criteria were adequately reported in 21 studies, and two lacked details on participant selection criteria (48, 52).

#### Classification criteria and assessment methods

The respondents in the included studies were classified into two to four broad groups according to the following different criteria, namely: (a) T2DM status (n=11) (5, 8, 13, 17, 18, 43, 45, 46, 50–52); (b) OSA severity or into OSA and non-OSA (n=8) (10–12, 14, 41, 42, 47, 49), or grouped two times by T2DM status and OSA status (n=2) (40, 44); (c) Subjective report of having or not having sleepiness (n=1) (39); and (d) According to body mass index (BMI) as normal BMI or elevated BMI (n=1) (48).

Assessment methods of OSA and T2DM are presented in **Table 3**. Objective methods were utilised in 15 studies to assess OSA, with four studies implementing clinic PSG and 11 studies utilising home portable sleep monitors (8, 10–14, 17, 18, 41, 47–52). In studies that used home portable sleep monitors, only three mentioned validating these home sleep monitors (18, 49, 51).

**Table 3:** Summary of methodological characteristics of the review studies.

Domain	Number (%)	Studies
Study design		
Cohort study	13 (56.5)	(8, 10, 12–14, 18, 39–44)
Cross-sectional	10 (43.5)	(5, 17, 45–52)
Sample characteristics		
Studies inclusion of both sexes	18 (78.2	(5, 8, 10, 12–14, 40–48, 50–52)
Studies included only male	4 (17.4)	(11, 17, 18, 49)
Studies consisted of male participants (male representation 60% or more)	5 (21.7)	(8, 10, 13, 40, 47)
Methodology characteristics		
Sample power calculation included	3 (13.0)	(5, 8, 13)
Comparator		
Matched control	2 (8.7)	(40, 43)
Unmatched control	21 (91.3)	(5, 8, 10–14, 17, 18, 39, 41, 42, 44–52)
Direction of association		
OSA increases the risk of T2DM	10 (43.5)	(8, 10–14, 18, 39, 41, 42).
T2DM increases the risk of OSA	1 (4.3)	(43)
Bidirectional association of OSA and T2DM	2 (8.6)	(40, 44)
Unspecified direction	10 (43.5)	(5, 17, 45–52)
Methods of assessment for OSA		
Objective OSA assessment (e.g., PSG or home portable sleep monitors)	15 (65.2)	(8, 10–14, 17, 18, 41, 47–52)
Subjective OSA assessment (e.g., sleep questionnaire)	5 (21.7)	(5, 39, 44–46)
Medical record codes for OSA	3 (13.0)	(40, 42, 43)
Standard objective assessment OSA scoring criteria	9 (39.1)	(8, 10, 13, 14, 41, 49–52)
Standard subjective assessment OSA scoring criteria	2 (6.8)	(46, 49)
Methods of assessment for T2DM		
Objective T2DM assessment (e.g., HbA1C)	17 (73.9)	(5, 8, 10–12, 14, 17, 18, 39, 45–52)
Subjective T2DM assessment (e.g., self-report)	2 (8.6)	(41, 44)
Medical record codes for T2DM	4 (17.4)	(13, 40, 42, 43)
Other methodological aspects		
Missing data treatment addressed	11 (47.8)	(5, 10, 13, 17, 39, 42, 43, 48–50, 52)
Attrition rate reported in cohort studies	6 (46)	(10, 12–14, 18, 41)
Sensitivity analysis conducted	10 (43)	(5, 8, 10, 13, 14, 39–41, 47, 52)
Adequate report of limitations and residual factors	13 (56.5)	(10–13, 17, 18, 41, 42, 45–47, 49, 50).

OSA, obstructive sleep apnea; T2DM, type 2 diabetes mellitus; PSG, polysomnography; HbA1C, haemoglobin A1C.

**Table 4** shows assessment methods, operational definitions, OSA and T2DM classification criteria and follow-up period for cohort studies. In studies that objectively evaluated OSA using clinic PSG and home portable sleep monitors, the apnea-hypopnea index was used to define OSA severity and classification following the criteria outlined by the American Academy of Sleep Medicine in 9 studies (53). However, six studies did not follow the standard criteria of the American Academy of Sleep Medicine for OSA classification (11, 12, 17, 18, 47, 48). Instead, these studies used different thresholds or combined moderate and severe categories in one group. Furthermore, three studies relied on medical records without providing clarification on assessment methods or classification criteria (40, 42, 43).

**Table 4:** Overview of diagnostic methods and operational definitions for OSA and T2DM of the review studies.

Author/year	OSA assessment method/duration	OSA criteria	T2DM assessment	T2DM criteria	Follow-up in months
1 KorshÃ¸j 2020 (49)	BQ, Pulse oximetry and nasal airflow for ≥4 h	BQ= high OSA risk (2 positive categories or 2 points in category 1 or 2 + BMI≥ 30kg/m2 or HTN). OSA= No OSA (AHI < 5/h), mild OSA (5/h ≤AHI < 15/h), moderate OSA (15/h ≤ AHI ≤ 30/h), severe OSA (AHI > 30/h)	Blood sample	HbA1C ≥ 48 mmol/L, random BG > 7 mmol/L	NA
2 Harada 2012 (17)	ESS, home portable machine recodes ≥ 2h	Mild OSA (RDI 5–14.9/hr), moderate OSA (RDI 15–29.9/hr), severe OSA (RDI ≥ 30/hr)	Blood sample and Self-report	FBG ≥ 126 mg/dL, IFG ≥ 110 mg dL and <126 mg dL, hypoglycaemic medication	NA
3 Lindberg 2012 (18)	ESS, home portable machine record for 1 night	ODI >5/hr, AHI>5/hr	Blood sample	FBG 77.0 mmol/L, BPG 6.1 to 7.0 mmol/L	136
4 Leong 2014 (50)	Home portable machine record for ≥4 h	Mild OSA (5/h ≤AHI < 15/h), moderate OSA (15/h ≤ AHI ≤ 30/h), severe OSA (AHI > 30/h)	Blood sample and Self-report	HbA1c ≥6.5%, physician referral or self-report, or hypoglycaemic medication	NA
5 Vacelet 2021 (12)	ESS, home portable machine record for ≥5 h	AHI ≥15/h	Blood sample	FBG ≥ 126 mg/d, HOMA-IR ≥ 2	84
6 Appleton 2015 (11)	Home portable machine record for ≥5.5 h	Mil OSA (10–19 AHI/h), moderate OSA (20–29 AHI/h), and severe OSA (≥ 30AHI/h)	Blood sample and Self-report	FBG ≥ 7.0 mmol/L, HbA1c of ≥ 6.5%, self-report of T2DM, or hypoglycaemic medication	56
7 Ding 2021 (8)	ESS, clinic PSG	No OSA (AHI < 5/h), mild OSA (5/h ≤AHI < 15/h), moderate OSA (15/h ≤ AHI ≤ 30/h), severe OSA (AHI > 30/h)	Blood sample and Self-report	FBG ≥ 126 mg/dL and new T2DM diagnosis	61
8 Deol 2018 (46)	BQ	High OSA risk 2 positive categories or 2 points in category 1 or 2 + BMI≥ 27.5 kg/m^2^ or HTN	Blood sample and Self-report	FBG ≥ 126 mg/dL, 2-h OGTT ≥ 200 mg/dL, or hypoglycaemic medication	NA
9 Sanchez 2022 (51)	ESS, home portable machine record for ≥3 h	No OSA (AHI < 5/h), mild OSA (5/h ≤AHI < 14.9/h), moderate OSA (15/h ≤ AHI ≤ 29.9/h), severe OSA (AHI ≥ 30/h)	Blood sample	HbA1c 5.7 to 6.4%	NA
10 Xu 2019 (10)	Clinic PSG	No OSA (AHI < 5/h), mild OSA (5/h ≤AHI < 14.9/h), moderate OSA (15/h ≤ AHI ≤ 29.9/h), severe OSA (AHI ≥ 30/h)	Blood sample and Self-report	FBG ≥ 7.0 mmol/L, OGTT ≥ 11.1 mmol/L, random BG ≥ 11.1 mmol/L, HbA1c of ≥ 6.5%, T2DM diagnosis, or hypoglycaemic medication	87
11 Ali 2023 (14)	Home portable machine with no record time	No OSA (AHI 0-4.5 5/h), mild OSA (5/h ≤AHI < 14.9/h), moderate to severe OSA (AHI ≥ 15/h)	Blood sample	FBG ≥ 126 mg/dL, 2-h OGTT ≥ 200 mg/dL	96
12 Kim 2013 (48)	Home portable machine with no record time	AHI ≥5	Blood sample	FBG ≥ 7.0 mmol/L, OGTT ≥ 11.1 mmol/L, HbA1c of ≥ 6.5%, T2DM diagnosis	NA
13 Nagayoshi 2016 (41)	Home portable machine with no record time	No OSA (AHI<5/h), mild OSA (AHI 5/h - 14.9/h), moderate OSA (AHI 15/h - 29.9/h), severe OSA (AHI ≥ 30/h)	Self-report	2DM diagnosis, or hypoglycaemic medication	156
14 Whitaker 2018 (52)	Home portable machine with no record time	Mild OSA (AHI 5/h - 14.9/h), moderate OSA (AHI 15/h - 29.9/h), severe OSA (AHI ≥ 30/h)	Blood sample	HbA1C	NA
15 Beate Strand 2015 (39)	Self-report OSA symptoms	Symptoms of observed apnea, bothersome snoring, and daytime sleepiness	Blood sample and Self-report	FBG ≥ 7.0 mmol/L, random BG ≥ 11.1 mmol/L, hypoglycaemic medication	61
16 Sabanayagam 2012 (5)	Self-report OSA symptoms	Symptoms of snoring, snorting, daytime sleepiness	Blood sample and Self-report	FBG ≥ 7.0 mmol/L, random BG ≥ 11.1 mmol/L, HbA1c of ≥ 6.5%, hypoglycaemic medication, T2DM diagnosis	NA
17 Kent 2014 (47)	ESS, polygraphy or clinic PSG	Mild OSA (ODI 5–14.9/hr), moderate OSA (ODI 15–29.9/hr), severe OSA (ODI ≥ 30/hr)	Blood sample	HbA1c of ≥ 6.5%	NA
18 D’Aurea 2017 (45)	BQ	High OSA risk 2 positive categories or 2 points in category 1 or 2 + BMI≥ 30kg/m2 or HTN	Blood sample and Self-report	IFG ≥ 110 mg/dL and <126 mg dL, HbA1c of ≥ 6.5%, hypoglycaemic medication. Prediabetes =HbA1c of 5.7%-6.4%	NA
19 Kendzerska 2014 (13)	ESS, clinic PSG	No OSA (AHI<5/h), mild OSA (AHI 5/h - 14.9/h), moderate OSA (AHI 15/h - 29.9/h), severe OSA (AHI ≥ 30/h)	Medical record	NR	67
20 Strausz 2018 (42)	Medical record	NR	Medical record	NR	264, 173, 300
21 Liu 2017 (40)	PSG (not indicated if clinic or home device)	NR	Medical record	NR	144
22 Tianyi 2018 (44)	Self-report OSA symptoms	NR	Self-report	Self-report of medication or self-report of IFG	168
23 Subramanian 2019 (43)	Medical record	NR	Medical record	NR	49

OSA, obstructive sleep apnea; T2DM, type 2 diabetes mellitus; BQ, Berlin Questionnaire; h, hour; BMI, body mass index; HTN, hypertension; AHI, apnea-hypopnea index; HbA1C, haemoglobin A1C; BG, blood glucose; ESS, Epworth Sleepiness Scale; RDI, respiratory desaturation index; FBG, fasting blood glucose; IFG, impaired fasting glucose; ODI, oxyhemoglobin desaturation index; PSG, polysomnography; OGTT, oral glucose tolerance test; NR, not recorded.

**Figure 3:**
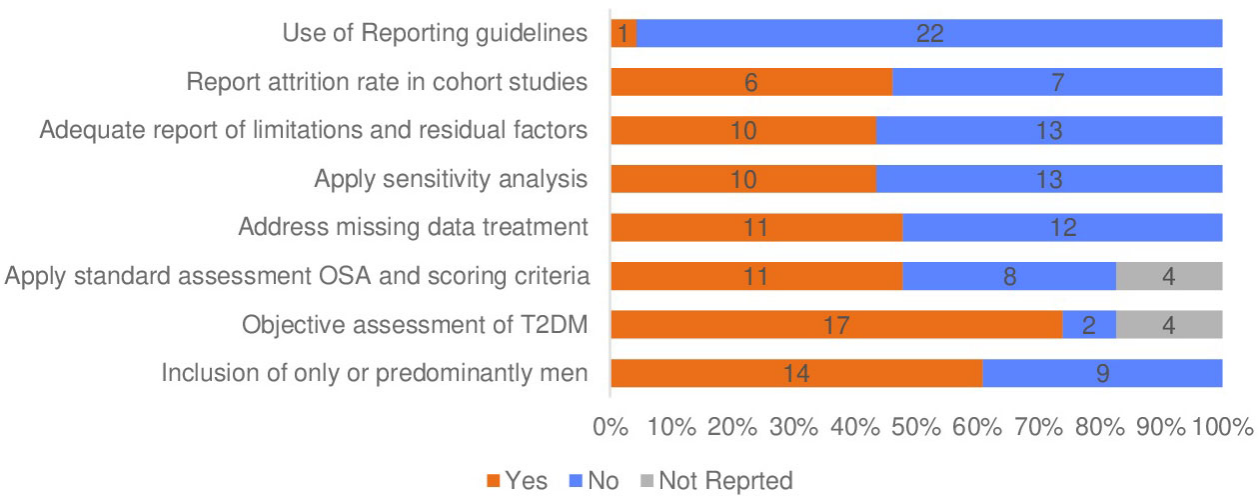
Key methodological features in the review studies.

Self-reporting was used to evaluate OSA in five studies, with two of them employing the validated Berlin questionnaire version (5, 39, 44–46). The remaining three studies either used modified versions of sleep questionnaires or questions about sleep quality (5, 39, 44). In three studies, in addition to self-reporting of OSA symptoms, medical records diagnoses, or codes were utilised to confirm OSA without specifying the methods of assessment or severity of OSA (40, 42, 44). In summary, 11 studies used standardised objective or subjective OSA assessment criteria (see **Table 4**, **Figure 3**).

Similarly, there was variation in the criteria used to diagnose T2DM across studies. Objective measurement using blood samples was applied in 17 studies (73.9%) (5, 8, 10–12, 14, 17, 18, 39, 45–52). Two studies used only self-reporting to confirm T2DM status (41, 44). The other four studies used medical records to confirm T2DM diagnosis (13, 42, 43, 54) (see **Table 4**).

#### Direction of hypothesis

Three primary hypotheses were identified in this review (see **Table 3**), with the majority of the cohort studies investigating whether OSA is linked to an increased risk of T2DM. In this review, ten cohort studies test the hypothesis that OSA increases the likelihood of developing T2DM or leads to diabetes variability (8, 10–14, 18, 39, 41, 42). One cohort study evaluated the opposite direction by assessing the risk of developing OSA among those with T2DM (43). Two cohort studies investigated a bidirectional association between OSA and T2DM (40, 44). Finally, as expected, the cross-sectional studies (n=10) did not indicate a specific direction of the association between OSA and T2DM but only hypothesised a relationship (5, 17, 45–52).

#### Comparator

A comparator group was deemed essential to meet the eligibility criteria outlined for this review. Two distinct types of comparator groups were identified, which are the control group and matched control group (see **Table 3**). Among the studies analysed in this review, the majority (21 out of 23) employed an unmatched control group, with two cohort studies utilising a matched control group for comparison (40, 43). In the first study by Liu, which tested bidirectional association, two approaches were used to assign the comparison subjects. To test the incidence of T2DM, ten non-OSA comparison subjects were randomly selected. They were matched by sex and year of birth, with no prior history of T2DM and equal cohort entry (40). In the same study, Liu matched two comparison subjects by sex, year of birth, and no prior history of OSA to test the risk of developing OSA in diabetic and non-diabetic subjects (40). The second study randomly assigned four control subjects matched by age, sex, and BMI to assess the incidence of developing OSA in diabetic and non-diabetic individuals (43).

#### Statistical methods

**Table 5** presents the primary statistical methods used namely: standard linear model, generalised linear model and survival analysis. Standard linear model was the most popular statistical method and included methods of a simple linear model, analysis of variance (ANOVA), and analysis of covariance (ANCOVA), and was applied in 52.17% (n=12) of the review studies (11, 12, 14, 17, 18, 39, 46, 48–52). The generalised linear model was applied in 47.8% (n=11) of the included studies (5, 11–14, 18, 40, 43, 45, 47, 48). The generalised linear model includes multiple logistic regression methods, stepwise multiple regression analyses, Poisson regression, and binomial regression. The survival analysis using the Kaplan-Meier method and Cox regression was applied in 34.78% (n=8) and included (8, 10, 13, 39–42, 44). Multilevel models fall under generalised linear regression, which is used to assess the changes toward developing T2DM within and between individuals using covariates. The Multilevel model was applied in one study using a series of nested models (41). Within the cohort of this review, 39.1% (n=9) applied more than one statistical method (11–14, 18, 39–41, 48).

**Table 5:** Primary statistical models in the review studies.

Statistical Methods	Number of studies n (%)	Publication
Standard Linear Model	12 (52.17)	(11, 12, 14, 17, 18, 39, 46, 48–52)
Generalised Linear Model	11 (47.8)	(5, 11–14, 18, 40, 43, 45, 47, 48)
Survival Analysis	8 (34.78)	(8, 10, 13, 39–42, 44)
Multilevel model	1 (4.0)	(41)
Two or more statistical methods	9 (39.13)	(11–14, 18, 39–41, 48)

**Table 2** shows the variation in the number of covariates that were introduced in the statistical model that may mediate the association between OSA and T2DM. The number of the tested covariates in the included studies ranged between 2-15 variables, with two studies lacking to adjust for important factors in the association, including age and BMI (44, 49). In more than half (56.5%, n=13) of the studies included, the report did not provide sufficient transparency about the limitations of the study and the potential residual factors that could impact the interpretation of the results (see **Table 3**) (10–13, 17, 18, 41, 42, 45–47, 49, 50).

#### Missing data and attrition rate

Within this review, 11 studies reported the method of handling missing data, and more than half of the studies (n=12) did not describe the methods used to manage missing data, as shown in **Table 3**. Methods of treating missing data captured in the included studies were complete case analysis (n=8), multiple imputation (n=3), missing indicator method (n=1) and last observation carried forward (n=1) (5, 10, 13, 17, 39, 42, 43, 48–50, 52). One longitudinal study employed three techniques to handle missing values, including multiple imputations for the covariate, excluding participants who were missing glucose and sleep symptoms data, and the last observation carried forward for time-dependent variables such as change in the waist circumference (39). The attrition rate was also underreported; nearly 54% (n=7) of cohort studies did not provide information about the lost cases, while data completeness report was missed in all studies (10, 12–14, 18, 41). Moreover, most studies did not indicate following the guidelines for presenting observational studies (55, 56). Only one study used the STROBE criteria to evaluate their reporting, but it was not fully adhered to in certain areas, such as reporting the reasons for missing data (**Figure 2**) (43). **Table 2** presents the quality assessment of the included studies. The critical appraisal showed four studies with fair quality (39, 45, 49, 50). While nineteen studies with good quality.

#### Sensitivity analysis

Out of the 23 studies reviewed, only 43% (n=10) of studies performed sensitivity analysis (see **Table 3**) (5, 8, 10, 13, 14, 39–41, 47, 52). All ten studies that performed sensitivity analysis satisfied the null hypothesis. However, the method of selecting the variables was unclear. Sensitivity analysis is an important step to strengthen causal inferences by measuring the uncertainty in the dependent variable based on the change due to input variation (57). In simpler terms, it examines how much the outcome changes depending on different values of a set of independent variables. Sensitivity analysis is performed to improve the accuracy and reliability of study results (57).

### Methodological issues of the review studies

This review highlights that the mixed results observed in studies investigating the relationship between OSA and T2DM may be driven by differences in methodological approaches, OSA classification methods, and study population characteristics (see **Tables 2**, **4**). The primary aim of the reviewed studies was to determine the frequency of OSA and T2DM or how each condition increases the risk of the other. This approach often relied on a single criterion, such as the apnea-hypopnea index (AHI) from polysomnography (PSG). However, these methods frequently overlooked significant differences between respondent subsets, including sociodemographic factors, which may account for the inconsistent findings. Notably, more than 52% of the review studies used non-standardized diagnostic criteria for assessing OSA.

Current variations in methodological approaches and sampling techniques identified in this review may not be sufficient to capture the health disparities among participants with OSA and T2DM. A standardised approach that includes comprehensive assessment methods for OSA and T2DM considers both sexes and incorporates a variety of sociodemographic and clinical factors may provide a more robust understanding of their association. By adopting a standardised approach, such as using an EDI (Equity, Diversity, and Inclusion) checklist, future research could systematically examine how various factors interact to influence the prevalence and interrelation of OSA and T2DM. This method ensures consistent data collection and analysis across studies, leading to more accurate and comparable results. Such a comprehensive approach has the potential to significantly advance our understanding of the relationship between OSA and T2DM, enabling the development of more targeted and effective interventions. Consequently, this could inspire and motivate further research in this field, ultimately improving clinical practice and public health outcomes.

## Discussion

In this review, we analysed 23 observational studies that explored the relationship between OSA and T2DM. This review aimed to examine the methodological approaches used by studies to establish the relationship between OSA and T2DM. We analysed the sampling techniques, sample characteristics, assessment methods, and statistical approaches employed to explore the association between OSA and T2DM. Previous systematic reviews have focused on estimating the prevalence and risk factors of OSA and T2DM but did not take into account the differences in population characteristics and assessment methods used to study OSA and T2DM (7, 22, 58). These differences can shed light on the varying rates and intensity of the reported connection (4).

The studies in our review involved different sample characteristics, sampling methods, operational definitions, geographic settings, and number of covariates. However, it is worth noting that there were significant discrepancies in the number of male and female participants across studies, with nearly half of the studies (n=9) consisting solely or mainly of male participants (8, 10, 11, 13, 17, 18, 40, 47, 49). The overrepresentation of males may limit the applicability of the results to the broader population, as gender-specific differences in disease prevalence, symptoms, and response to treatments are well-documented in medical literature (59–61). Therefore, findings predominantly derived from male participants may not fully capture the nuances and variations that could exist among female populations. These differences in sample characteristics and participant selection could substantially impact our comprehension of the correlation between OSA and T2DM. Moreover, the studies categorised all participants into broad groups without considering individual nuances. This could lead to an insufficient understanding of health disparities and impede the diagnosis and management of OSA patients in clinical settings. In line with our findings, Tahrani and Ali (4) indicated in their report that the variation of participants’ characteristics might explain the differences in the reported strength of association between OSA and T2DM.The current review studies primarily utilised administrative and previously conducted study data to address research inquiries. However, only two studies employed primary data for the purpose of the studies (17, 49). Administrative data uses a non-probability convenience sampling technique that has both benefits and drawbacks (35). One of its most significant advantages is its cost-effectiveness and ease of collecting data quickly (62). However, the non-probability sampling method may have resulted in biased findings, leading to an overestimation of the prevalence of a health condition (62, 63). Therefore, an inaccurate representation of the population challenges the generalisation of the results to the entire population (62). Thus, researchers using secondary datasets, such as national surveys, should perform an adequate assessment of the used dataset to identify the quality of the collected data and its limitations, sampling methods to limit the source of bias and enhance the validity of the findings (62, 64). Researchers should also take note of the differences between the control and disease groups and adjust their statistical analysis accordingly to avoid misleading conclusions (64).

This review identified two study designs: Cross-sectional studies (n=10) and cohort studies (n=13). Cross-sectional studies aimed to examine the correlation between OSA and T2DM, but this design has methodological and statistical limitations. For instance, it cannot determine the direction of the association and causal inferences due to the lack of temporal elements (34). Alternatively, cohort studies can identify and relate an outcome to a specific exposure after a follow-up period, suggesting a cause-effect relationship (34). Among the thirteen cohort studies, six were retrospective, and seven were prospective. The prospective cohort design is superior to the retrospective cohort approach, which is subject to recall bias, and the concurrent presence of two diseases at baseline cannot be rolled out (34). Similar to cross-sectional design, cohort studies also have limitations, such as the risk of participant drop-out, which may affect the validity and generalisability of the results (34). Therefore, it is recommended that sufficient information about drop-outs be provided and sensitivity analysis performed to compare the characteristics of drop cases with completed cases to enhance the study’s validity (57). In this review, around 54% of the cohort studies did not provide details about dropped cases, and no study performed a sensitivity test to compare the characteristics of dropped cases with complete cases (10, 12–14, 18, 41).

The review studies lacked sufficient information on missing data treatment methods and utilisation of reporting guidelines. Most of the review studies (n = 21) used convenience samples, which means they used data collected for previous studies or administrative data. This approach may have resulted in incomplete cases being excluded from the analysis, leading to selection bias (64). Handling missing data was reported in 11 studies, but they did not provide enough detail on the methods used to address the missing data (5, 10, 13, 17, 39, 42, 43, 48–50, 52). The reader needs to obtain a comprehensive report of missing data and data completeness to evaluate the validity of study results and identify the source of bias (64). Furthermore, the majority of the studies included in our analysis did not report the study using guidelines such as STOBE or RECORD (55, 56). One cohort study reported on the adequacy of their study report using the STROBE checklist, but it was incomplete (43).

In this review, we observed inconsistencies in the operational definitions of OSA and T2DM across the included studies (**Table 4**). This variation was also observed in previous systematic reviews of studies that examined the association between OSA and T2DM (7, 58). In this review, 15 studies used PSG to diagnose OSA, six did not follow the American Academy of Sleep Medicine standard criteria for OSA classification and three studies relied on medical records without clarifying the classification criteria for OSA and T2DM (53). The variation was observed in the OSA classification criteria and severity cut-off values (see **Tables 2** **, 4**).

Additionally, in studies that used subjective methods to evaluate OSA (n=5), three studies did not report OSA using a standardised self-report checklist, such as Berlin Questionnaire (5, 39, 44–46, 65). Instead, these studies reported OSA based on self-reporting different sleep symptoms, such as snoring (5, 39). Similar inconsistencies were observed in defining T2DM, with some studies (n=17) using objective criteria (5, 8, 10–12, 14, 17, 18, 39, 45–52). On the other hand, two studies were based only on self-report to confirm T2DM (41, 44). Last, four studies used medical records to confirm T2DM (13, 42, 43, 54). These discrepancies in the operational definitions may have caused the variation in the reported results.The studies in this review mainly relied on PSG data that were reduced to one metric value of the apnea-hypopnea index or subjective sleep responses to reflect OSA status. Binarised diabetes parameters were used to test the correlation between OSA and T2DM. However, depending on a single parameter, such as the apnea-hypopnea index, does not capture the extensive range of differences in OSA signs and symptoms, demographic, physiological, and clinical characteristics (66). One of the current review studies by Ding and colleagues utilised PSG phenotyping to classify OSA participants and examine their vulnerability to chronic diseases, including T2DM (8). However, similar to other included studies, the study solely focused on PSG parameters and did not take into account other clinical and sociodemographic factors during the classification process. For a more comprehensive assessment of the correlation between OSA and T2DM, it may be helpful to consider an alternative method that takes into account demographic factors, clinical symptoms, and physiological effects. This approach could provide a more accurate assessment of the correlation between OSA and T2DM (66, 67).

### Recommendations for future research

In addition to exploring the methodological aspects of the studies examining the association between OSA and T2DM, we aimed to provide recommendations for future research. We identified variations between the included studies in terms of sample characteristics, with some studies predominantly including male participants, assessment tools, and classification criteria, which make result synthesis on the association between OSA and T2DM difficult. The associations between OSA and T2DM vary significantly depending on age, sex, and race (22). Therefore, we strongly suggest applying standardised assessment criteria for OSA and T2DM, incorporating more women, including diverse ethnicities, and using an EDI checklist to better understand the heterogeneity in OSA and to create equitable and personalised treatment approaches. Adopting these recommendations could lead to more specific diagnoses and individualised treatment approaches, potentially improving patient outcomes (66, 67).

There were also identified limitations in the report of the included studies, with several studies not providing sufficient details on missing data and attrition rates, which may affect the credibility of the results (64). Therefore, we recommend providing adequate details on sample characteristics, missing data, the approach used to deal with missing data, and dropped-out cases. Furthermore, we recommend performing sensitivity analyses to provide details of nonresponse cases in terms of characteristics compared to complete cases to eliminate bias related to the response rate (57). Finally, to ensure sufficient and reproducible research studies, we strongly encourage the use of reporting guidelines to improve the accuracy and transparency of reporting observational studies (55, 56).

### Strengths and limitations of the review

In contrast to previous reviews that examined OSA and T2DM, this review is the first systematic review that examined aspects of the methodology used to understand the association between OSA and T2DM. A second strength of this review is the examination of a large number of abstracts (n=3718) identified from six different databases. Furthermore, we updated the search to recognise recently published papers in July 2023 before starting data synthesis. A number of limitations were identified in our systematic review. Although we manually searched the bibliography, we may have missed relevant studies if OSA and T2DM were not detected in the titles of the included studies when searching their reference lists. Due to the heterogeneity of the included studies, we were unable to conduct a meta-analysis. Nevertheless, we attempted to provide a narrative analysis and extensive discussion on the methods applied in the review studies and how these methods influence our understanding of the association between OSA and T2DM. Additionally, our review was limited to English-language studies, which could introduce bias by excluding significant research from non-English-speaking regions. This limitation may affect the generalisability of our findings due to variations in healthcare systems, diagnostic criteria, and cultural contexts. Future research should include non-English language studies, employ multi-lingual and culturally diverse research teams, and consider cultural differences in study designs and analyses to enhance the comprehensiveness and applicability of findings on a global scale.

## Conclusion

This review examined 23 studies exploring the connection between OSA and T2DM, highlighting significant methodological variations such as differences in population characteristics, sampling procedures, and assessment tools. These variations limit our understanding of the factors that may be driving this correlation. By critically assessing these methodological aspects, we identified both the strengths and limitations of the included studies.

Our key findings suggest that future research should adopt standardised diagnostic criteria and comprehensive assessment methods, incorporate an EDI checklist, and consider a broad range of sociodemographic and clinical factors. Such an approach would enhance the accuracy and generalisability of findings, thereby providing a clearer understanding of the relationship between OSA and T2DM.

In clinical settings, implementing these recommendations could improve the identification and management of patients with OSA and T2DM, leading to more personalised and effective treatment strategies. Future research adhering to these enhanced methodological standards will not only fill existing knowledge gaps but also pave the way for improved clinical practices and better health outcomes for individuals suffering from OSA and T2DM.

## Data Availability

The original contributions presented in the study are included in the article/**Supplementary Material**. Further inquiries can be directed to the corresponding author.
