# Supplementary material for: A systematic review of the methodology for examining the relationship between obstructive sleep apnea and type two diabetes mellitus
Source: Front Endocrinol (Lausanne). 2024 Sep 4;15:1373919. doi: 10.3389/fendo.2024.1373919 (PMC11411564; doi:10.3389/fendo.2024.1373919)
Supplement: Supplementary file 1 [file Table1.docx]

**Appendix A**

# **A Systematic Review of the Methodology for Examining the Relationship Between Obstructive Sleep Apnea and Type Two Diabetes Mellitus**

Manal Taimah^1&2*^, Nirmin F. Juber^2^, Paula Holland^1^, and Heather Brown^1^

^1^Division of Health Research, Lancaster University, Lancaster, United Kingdom

^2^Public Health Research Center, New York University Abu Dhabi, United Arab Emirates

**Exact search terms on the database**

The terms were located and extracted from different sources including previous systematic reviews, research papers on the area of OSA and T2DM, and abstract keywords. The variable terms were listed and checked on the following database: MEDLINE, APA PsycINFO, and CINAHL (searched through the EBSCO database), Scopus, Web of Science Core Collection and EMBASE.

| **Medline Complete via EBSCO** | | |
| --- | --- | --- |
| **Search #** | **Search Terms** | **Results** |
| S1 | ( (MH "Sleep Apnea Syndromes+") OR (MH "Sleep Apnea, Obstructive+") OR (MH "Apnea") OR (MH "Disorders of Excessive Somnolence") ) OR TI ( "obstructive sleep apnea*" OR SDB OR SAHS OR "apnea hypopnea syndrome*" OR "OSAS" OR "Breathing Sleep-Disorder*" ) OR AB ( "obstructive sleep apnea*" OR SDB OR SAHS OR "apnea hypopnea syndrome*" OR "OSAS" OR "Breathing Sleep-Disorder*" ) | 73,986 |
| S2 | ( (MH "Diabetes Mellitus") OR (MH "Diabetes Mellitus, Type 2") OR (MH "Prediabetic State") OR (MH "Hyperglycemia") OR (MH "Insulin Resistance") OR (MH "Hyperinsulinism") ) OR TI ( diabetes OR diabetic OR Hyperinsulinism OR Hyperglyc* OR "Insulin Resistance" OR "Glucose Intolerance" OR Prediabet* ) OR AB ( diabetes OR diabetic OR Hyperinsulinism OR Hyperglyc* OR "Insulin Resistance" OR "Glucose Intolerance" OR Prediabet* ) | 878,914 |
| S3 | ( (MH "Epidemiologic Research Design") OR (MH "Research Design") OR (MH "Cross-Sectional Studies") OR (MH "Cohort Studies") OR (MH "Longitudinal Studies") ) OR TI ( "longitudinal stud*" OR "cohort stud*" OR "observational stud*" OR "case control stud*" OR "cross sectional stud*" OR incidence OR prevalence ) ) OR AB ( "longitudinal stud*" OR "cohort stud*" OR "observational stud*" OR "case control stud*" OR "cross sectional stud*" OR incidence OR prevalence ) ) | 2,832,183 |
| S4 | (MH "Adult+") OR TI adult* OR AB adult* | 8,625,158 |
| S5 | S1 & S2 & S3 & S4 | 1,206 |

| **APA PsycINFO** |  |  |
| --- | --- | --- |
| **Search #** | **Query** | **Results** |
| S1 | ( (DE "Sleep Apnea" OR DE "Sleep-Related Hypoventilation" OR "obstructive sleep apnea*" OR SDB OR SAHS OR "apnea hypopnea syndrome*" OR "OSAS" OR "Breathing Sleep-Disorder*") OR (DE "Sleep Wake Disorders") ) OR TI ( "obstructive sleep apnea*" OR SDB OR SAHS OR "apnea hypopnea syndrome*" OR "OSAS" OR "Breathing Sleep-Disorder*" ) OR AB ( "obstructive sleep apnea*" OR SDB OR SAHS OR "apnea hypopnea synd | 19,647 |
| S2 | ( ((DE "Diabetes" OR DE "Type 2 Diabetes") OR (DE "Diabetes Mellitus" OR DE "Type 2 Diabetes")) OR (DE "Hyperglycemia") OR diabet* OR diabetic OR Hyperinsulinism OR Hyperglyc* OR "Insulin Resistance" OR "Glucose Intolerance" OR Prediabet* ) OR TI ( diabet* OR diabetic OR Hyperinsulinism OR Hyperglyc* OR "Insulin Resistance" OR "Glucose Intolerance" OR Prediabet* ) OR AB ( diabet* OR diabetic OR Hyperinsulinism OR Hyperglyc* OR "Insulin Resistance" OR "Glucose Intolerance" OR Prediabet* ) | 43,062 |
| S3 | ( DE "Epidemiology" OR "Research Design" OR "longitudinal stud*" OR "cohort stud*" OR "observational stud*" OR "case control stud*" OR "cross sectional stud*" OR incidence OR prevalence ) OR TI ( "Research Design" OR "longitudinal stud*" OR "cohort stud*" OR "observational stud*" OR "case control stud*" OR "cross sectional stud*" OR incidence OR prevalence ) OR AB ( "Research Design" OR "longitudinal stud*" OR "cohort stud*" OR "observational stud*" OR "case control stud*" OR "cross sectional stud*" OR incidence OR prevalence ) | 470,345 |
| S4 | S1 AND S2 AND S3 | 285 |
| S5 | adult* OR TI adult* OR AB adult* | 1,080,406 |
| S6 | S4 AND S5 | 143 |

| **CINAHL** | | |
| --- | --- | --- |
| **Search #** | **Search Terms** | **Actions** |
| S1 | ( (MH "Sleep Apnea, Obstructive") OR (MH "Sleep Apnea Syndromes+") ) OR TI ( ( "obstructive sleep apnea*" OR SDB OR SAHS OR "apnea hypopnea syndrome*" OR "OSAS" OR "Breathing Sleep-Disorder*" ) ) OR AB ( ( "obstructive sleep apnea*" OR SDB OR SAHS OR "apnea hypopnea syndrome*" OR "OSAS" OR "Breathing Sleep-Disorder*" ) ) | 21,577 |
| S2 | ( (MH "Diabetes Mellitus, Type 2") OR (MH "Diabetes Mellitus") OR (MH "Prediabetic State") OR (MH "Hyperglycemia") OR (MH "Hyperinsulinism") OR (MH "Insulin Resistance") ) OR TI ( ( diabetes OR diabetic OR Hyperinsulinism OR Hyperglyc* OR "Insulin Resistance" OR "Glucose Intolerance" OR Prediabet* ) ) OR AB ( ( diabetes OR diabetic OR Hyperinsulinism OR Hyperglyc* OR "Insulin Resistance" OR "Glucose Intolerance" OR Prediabet* ) ) | 290,949 |
| S3 | ( (MH "Epidemiological Research") OR (MH "Study Design") OR (MH "Cross Sectional Studies") OR (MH "Prospective Studies") ) OR TI ( ( "longitudinal stud*" OR "cohort stud*" OR "observational stud*" OR "case control stud*" OR "cross sectional stud*" OR incidence OR prevalence ) ) OR AB ( ( "longitudinal stud*" OR "cohort stud*" OR "observational stud*" OR "case control stud*" OR "cross sectional stud*" OR incidence OR prevalence ) ) | 1,199,161 |
| S4 | S1 AND S2 AND S3 | 648 |
| S5 | (MH "Adult+") OR TI adult* OR AB adult* | 2,222,641 |
| S6 | S4 AND S5 | 427 |

| **Scopus** | | |
| --- | --- | --- |
| **Search #** | **Search query** | **Results** |
| 1 | TITLE-ABS-KEY ( "obstructive sleep apnea*" OR sdb OR sahs OR "apnea hypopnea syndrome*" OR "osas" OR "breathing sleep-disorder*" ) | [47,181](https://www-scopus-com.ezproxy.lancs.ac.uk/search/history/results.uri?origin=searchhistory&shid=14) |
| 2 | TITLE-ABS-KEY ( diabet* OR diabetic OR hyperinsulinism OR hyperglyc* OR "insulin resistance" OR "glucose intolerance" OR prediabet* ) | [1,328,032](https://www-scopus-com.ezproxy.lancs.ac.uk/search/history/results.uri?origin=searchhistory&shid=15) |
| 3 | TITLE-ABS-KEY ( "research design" OR "longitudinal stud*" OR "cohort stud*" OR "observational stud*" OR "case control stud*" OR "cross sectional stud*" OR incidence OR prevalence ) | [4,099,791](https://www-scopus-com.ezproxy.lancs.ac.uk/search/history/results.uri?origin=searchhistory&shid=16) |
| 4 | ( TITLE-ABS-KEY ( "obstructive sleep apnea*" OR sdb OR sahs OR "apnea hypopnea syndrome*" OR "osas" OR "breathing sleep-disorder*" ) ) AND ( TITLE-ABS-KEY ( diabet* OR diabetic OR hyperinsulinism OR hyperglyc* OR "insulin resistance" OR "glucose intolerance" OR prediabet* ) ) AND ( TITLE-ABS-KEY ( "research design" OR "longitudinal stud*" OR "cohort stud*" OR "observational stud*" OR "case control stud*" OR "cross sectional stud*" OR incidence OR prevalence ) ) | 2,535 |
| 5 | ( TITLE-ABS-KEY ( adult* ) ) AND ( ( TITLE-ABS-KEY ( "obstructive sleep apnea*" OR sdb OR sahs OR "apnea hypopnea syndrome*" OR "osas" OR "breathing sleep-disorder*" ) ) AND ( TITLE-ABS-KEY ( diabet* OR diabetic OR hyperinsulinism OR hyperglyc* OR "insulin resistance" OR "glucose intolerance" OR prediabet* ) ) AND ( TITLE-ABS-KEY ( "research design" OR "longitudinal stud*" OR "cohort stud*" OR "observational stud*" OR "case control stud*" OR "cross sectional stud*" OR incidence OR prevalence ) ) ) | 1,644 |

| **Web of Science Core Collection** | | |
| --- | --- | --- |
| **Search #** | **Search Query** | **Results** |
| 1 | TS=( "obstructive sleep apnea*" OR SDB OR SAHS OR "apnea hypopnea syndrome*" OR "OSAS" OR "Breathing Sleep-Disorder*" ) | 49322 |
| 2 | TS=(diabet* OR diabetic OR Hyperinsulinism OR Hyperglyc* OR "Insulin Resistance" OR "Glucose Intolerance" OR Prediabet* ) | 1067343 |
| 3 | TS=("Research Design" "longitudinal stud*" OR "cohort stud*" OR "observational stud*" OR "case control stud*" OR "cross sectional stud*" OR incidence OR prevalence ) | 2628991 |
| 4 | #1 AND #2 AND #3 | [1913](https://www-webofscience-com.proxy.library.nyu.edu/wos/woscc/summary/f21a2abe-f262-4bef-93ec-87dc39c4817f-7a21c20d/relevance/1) |
| 5 | TS=(adult*) | 2032419 |
| 6 | #4 AND #5 | 495 |

| **Embase Database** | | |
| --- | --- | --- |
| **Search #** | **Searches** | **Results** |
| 1 | Sleep Apnea/ or obstructive sleep apnea.mp. or Sleep Apnea Syndrome.mp. or apnea hypopnea syndrome.mp. or apnea-hypopnea syndrome.mp. or Sleep apnoea* hypopnoea.mp. or apnoea hypopnoea.mp. or Breathing Sleep-Disorder.mp. | 101539 |
| 2 | non insulin dependent diabetes mellitus/ or diabetes mellitus/ or exp Type 2 Diabetes/ or hyperglycem*.mp. or hyperinsulin*.mp. or exp Type 2 Diabetes/ or Prediabet* State.mp. or type-2 diabetes.mp. or diabetes.mp. or diabetic.mp. or Hyperinsulin*.mp. or Hyperglyc*.mp. or "Insulin Resistance".mp. or Prediabet*.mp. | 1551165 |
| 3 | (longitudinal or case-control or case control or cross-sectional stud* or cross sectional stud* or cohort stud* or longitudinal or follow up stud* or follow-up study or case control study or observational stud*).mp. | 2086972 |
| 4 | exp adult/ | 10639341 |
| 5 | 1 and 2 and 3 and 4 | 2190 |
