# Supplementary material for: A systematic review of the methodology for examining the relationship between obstructive sleep apnea and type two diabetes mellitus
Source: Front Endocrinol (Lausanne). 2024 Sep 4;15:1373919. doi: 10.3389/fendo.2024.1373919 (PMC11411564; doi:10.3389/fendo.2024.1373919)
Supplement: Supplementary file 2 [file Table2.doc]

**Appendix B**

# A Systematic Review of the Methodology for Examining the Relationship Between Obstructive Sleep Apnea and Type Two Diabetes Mellitus

Manal Taimah^1*^, Nirmin F. Juber^2^, Paula Holland^1^, and Heather Brown^1^

**Quality Assessment Tools**

**Newcastle - Ottawa quality assessment scale for cohort studies**

Reviewer: M.T Date:25^th^ June 2023

Author: Ali,T.Siddiquee Record Number: #6203

**Selection**

1) Representativeness of the exposed cohort

a) truly representative of the average _______________ (describe) in the community *****

- **b) somewhat representative of the average ______________ in the community ***

c) selected group of users eg nurses, volunteers

d) no description of the derivation of the cohort

2) Selection of the non exposed cohort

- **a) drawn from the same community as the exposed cohort ***

b) drawn from a different source

c) no description of the derivation of the non exposed cohort

3) Ascertainment of exposure

- **a) secure record (eg surgical records) ***

b) structured interview *****

c) written self report

d) no description

4) Demonstration that outcome of interest was not present at start of study

- **a) yes ***

b) no

**Comparability**

1) Comparability of cohorts on the basis of the design or analysis

- **a) study controls for _____________ (select the most important factor) ***

b) study controls for any additional factor *****

(This criteria could be modified to indicate specific control for a second important factor.)

**Outcome**

1) Assessment of outcome

a) independent blind assessment *****

- **b) record linkage ***

c) self report

d) no description

2) Was follow-up long enough for outcomes to occur

- **a) yes (select an adequate follow up period for outcome of interest) ***

b) no

3) Adequacy of follow up of cohorts

a) complete follow up - all subjects accounted for *****

b) subjects lost to follow up unlikely to introduce bias - small number lost - > ____ % (select an adequate %) follow up, or description provided of those lost) *****

- **c) follow up rate < ____% (select an adequate %) and no description of those lost**

d) no statement

**Judgment:**

Thresholds for converting the Newcastle-Ottawa scales to AHRQ standards (good, fair, and poor):

Good quality: 3 or 4 stars in selection domain AND 1 or 2 stars in comparability domain AND 2 or 3 stars in outcome/exposure domain

Fair quality: 2 stars in selection domain AND 1 or 2 stars in comparability domain AND 2 or 3 stars in outcome/exposure domain

Poor quality: 0 or 1 star in selection domain OR 0 stars in comparability domain OR 0 or 1 stars in outcome/exposure domain

**Total number of stars: 8**

**Overall study quality:**

- **a) good quality**

b) Fair quality

c) Poor quality

Additional notes: Include the study, the study showed good quality with adequate explanation of inclusion and exclusion criteria and adequate follow-up period (57.5 ± 5.5). the study control for important confounding factors.

JBI critical appraisal checklist for analytical cross-sectional studies

Reviewer: N.J Date:15^th^ May 2023

Author: Korshøj 2020 Record: Number: #3249

|  | Yes | No | Unclear | Not applicable |
| --- | --- | --- | --- | --- |
| 1. Were the criteria for inclusion in the sample clearly defined? |  | □ | □ | □ |
| 1. Were the study subjects and the setting described in detail? |  | □ | □ | □ |
| 1. Was the exposure measured in a valid and reliable way? |  | □ | □ | □ |
| 1. Were objective, standard criteria used for measurement of the condition? | □ |  | □ | □ |
| 1. Were confounding factors identified? | □ |  | □ | □ |
| 1. Were strategies to deal with confounding factors stated? Not included important confounding factors | □ |  | □ | □ |
| 1. Were the outcomes measured in a valid and reliable way? |  | □ | □ | □ |
| 1. Was appropriate statistical analysis used? |  | □ | □ | □ |

Overall appraisal: Include **√** Exclude □ Seek further info □

Comments (Including reason for exclusion)

The study included a small sample of participant of truck drivers, and no women were included. The study used non standardized assessment criteria for OSA and no adjustment for important confounding factors such as obesity, gender.
