# Supplementary material for: A systematic review of the methodology for examining the relationship between obstructive sleep apnea and type two diabetes mellitus
Source: Front Endocrinol (Lausanne). 2024 Sep 4;15:1373919. doi: 10.3389/fendo.2024.1373919 (PMC11411564; doi:10.3389/fendo.2024.1373919)
Supplement: Supplementary file 4 [file Table4.docx]

# **Appendix D**

# **A Systematic Review of the Methodology for Examining the Relationship Between Obstructive Sleep Apnea and Type Two Diabetes Mellitus**

Manal Taimah^1&2*^, Nirmin F. Juber^2^, Paula Holland^1^, and Heather Brown^1^

^1^Division of Health Research, Lancaster University, Lancaster, United Kingdom

^2^Public Health Research Center, New York University Abu Dhabi, United Arab Emirates

**Heterogeneity Analysis**

Supplementary Table 1 provides data on the effect sizes and their confidence intervals extracted from each included study. Depending on the study, the effect sizes were reported as odds ratios, beta values, and hazard ratios. The following studies were included in the analysis:

**Supplementary Table1**

| **Author/Year** | **Effect size** | **Lower Confidence Interval** | **Upper Confidence Interval** | **Effect Type** |
| --- | --- | --- | --- | --- |
| KorshÃ¸j 2020 | 1.83 | 0.67 | 8.29 | Odds ratio |
| Harada 2012 | 0.45 | 0.45 | 19.9 | Beta coefficient |
| Lindberg 2012 | 4.2 | 0.9 | 18.3 | Odds ratio |
| Leong 2014 | 0.0011 | 0.0088 | 0.0109 | Beta coefficient |
| Vacelet 2021 | 1.97 | 0.84 | 4.63 | Odds ratio |
| Appleton 2015 | 2.6 | 1.1 | 6.1 | Odds ratio |
| Ding 2021 | 3.18 | 1.52 | 6.66 | Hazards ratio |
| Sanchez 2022 | 2.20 | 0.10 | 4.31 | Beta coefficient |
| Xu 2019 | 2.62 | 1.4 | 4.93 | Hazards ratio |
| Ali 2023 | 1.5 | 1.02 | 2.21 | Relative risk |
| Kim 2013 | 1.7 | 1.07 | 2.69 | Odds ratio |
| Nagayoshi 2016 | 2.03 | 1.2 | 3.44 | Hazards ratio |
| Whitaker 2018 | 7.25 | NA | NA | Adjusted mean |
| Beate Strand 2015 | 2 | 1.35 | 2.98 | Hazards ratio |
| Sabanayagam 2012 | 2.04 | 1.46 | 2.87 | Odds ratio |
| Kent 2014 | 1.87 | 1.45 | 2.42 | Odds ratio |
| D'Aurea 2017 | 1.61 | 1.19 | 2.2 | Odds ratio |
| Kendzerska 2014 | 1.06 | 0.99 | 1.13 | Hazards ratio |
| Strausz 2018 | 1.48 | 1.26 | 1.73 | Hazards ratio |
| Liu 2017 | 1.33 | 1.22 | 1.46 | Hazards ratio |
| Tianyi 2018 | 1.37 | 1.24 | 1.53 | Hazards ratio |
| Subramanian 2019 | 1.31 | 1.22 | 1.4 | incidence rate |

Summary of Effect Sizes and Confidence Intervals for the Review Studies

**The heterogeneity among studies was assessed using Cochran's Q test, with significance set at p < 0.05.** Effect sizes reported across studies were standardized to a common metric odds ratio (OR) to account for variations in study designs and measurements. To assess heterogeneity, we conducted the following tests:

1. **Cochran's Q Test**: This test was used to evaluate whether observed variations in effect sizes across studies were greater than expected by chance.
2. **I² Statistic**: This statistic quantifies the proportion of total variation in study estimates that is due to heterogeneity rather than sampling error.
3. **Tau² Estimate**: This provides an estimate of the between-study variance.

**Heterogeneity Test Results**

Supplementary Table 1 shows the heterogeneity test results. The heterogeneity analysis revealed significant variability among the included studies. The Cochran's Q test was significant (Q = 122.99, df = 20, p < 0.001), indicating more variability in effect sizes than expected by chance. The I² statistic showed that 83.7% of the total variation was due to heterogeneity (95% CI: 9.5% to 93.5%), suggesting substantial heterogeneity among the studies. The H value of 2.48 (95% CI: 1.051 to 3.929) and the tau² estimate of 0.0445 further support the presence of significant heterogeneity.

These results highlight the impact of methodological differences, such as variations in study design, diagnostic criteria, and participant characteristics, on the reported associations between obstructive sleep apnea (OSA) and type 2 diabetes mellitus (T2DM). Addressing these variations is crucial for improving the consistency and reliability of future research findings in this area.

**Supplementary Table 2**

Heterogeneity Test Results for the review studies

| Measure | Value |
| --- | --- |
| Cochran's Q | 122.99 |
| P value | 0.000 |
| df | 20 |
| H | 2.480 (95%CI: 1.051 to 3.929) |
| I² (%) | 83.7% (95%CI: 9.5% to 93.5% |
| tau² | 0.0445 |
| H = relative excess in Cochran's Q over its degrees of freedom.  I² = proportion of total variation in effect estimate due to between-study heterogeneity (based on Q).  Abbreviations: df, degree of freedom; CI, confidence interval | |
